# The Story of the Insane from Year to Year

**Published:** 1893-01-21

**Authors:** 


### THE STORY OF THE INSANE FROM YEAR TO YEAR.

(FIFTH SERIES.)

### CITY OF LONDON ASYLUM.

This asylum is situated at Stone, near Dartford, in Kent. It is a small asylum, containing only 386 patients on the last day of 1891. The admissions during the year amounted to fifty, the recoveries to twenty-five, and the deaths to nineteen. The percentage of recoveries was far above the average of English county asylums, and still further above their own average. The former is about 40 per cent., the latter 30·68, whereas the percentage for 1891 was 50. The death-rate is extremely low, being only 4 88 on the average number resident. As a rule the death-rate depends more on the character and frequency of the admissions than on the sanitary state of the asylum, it would seem clear, in spite of the fatal case of enteric fever, that the asylum is in a satisfactory state. Of the total of 386, only fourteen were believed to be curable cases. No fewer than twenty of the admissions were caused by drink, but we notice that only four were owing to hereditary influence. It has been decided to admit private patients at this asylum, and very useful instructions concerning certificates, &c., are given on page 16 of the report. The Committe and the Medical Superintendent are to be congratulated on this step, but we think the weekly rate of payment ought to have been less than a guinea. It is commendable that Dr. White finds time amid his numerous duties to lecture to the attendants and nurses, and he is able to point to useful results—always an invigorating thing to anyone who has taken pains to instruct—for we notice that his head attendant, head nurse, and ten of the staff have passed examinations in the subjects taught. Dr. White thinks the absence of bed-sores is owing to the theoretical and practical training enjoyed by the staff. That there is something in this we gladly allow; but Dr. White will find, what others have found before him, that these sores cannot be altogether prevented. Many structural improvements have been carried out during the year. The Visitors say "the Medical Superintendent and the officers generally have discharged their duties so as to merit a continuance of the confidence placed in them." There is a balance of £254 in favour of the farm, but we do not see anything charged for rent of land, interest on capital, or patients' labour. The conclusions are therefore valueless for the sake of comparison. We notice no table showing the weekly cost per head, nor did we see any incidental mention of the cost in any of the reports.

### HOLLOWAY SANATORIUM.

This is a registered hospital for the insane, built at Virginia Water by the late Thomas Holloway. It now contains 327 patients, although the report under notice shows that this is only the sixth complete year of its existence. It was opened in June, 1885, and on the last day of that year it had only 62 patients. During 1891 the admissions reached 156; the recoveries were 67, and the deaths 21. Thrown into percentages, these numbers yield 54·9 recoveries and 6·6 deaths; the first being calculated on the total number of admissions, and the second on the average number resident. Hereditary tendency was the ascertained cause in only 26 cases; but as Dr. Philipps well remarks, "it is notoriously difficult to arrive at the truth on this point." Twelve cases are supposed to have been caused by influenza or other specific fever. The admissions were composed of 68 men and 88 women, and Dr. Philipps shows that although the liability to insanity seems greater with women than with men, their chances of recovery are also greater, being 43·7 per cent, as against 35·2 per cent. It is well known that the Sanatorium, when handed over to the committee and Medical Superintendent, had many structural defects. Some of the more glaring of these were speedily altered under Dr. Philipps' direction, and every year has seen minor alterations and improvements carried out. It is required by the rules of this hospital that at least one-fourth of the patients shall pay less than twenty-five shillings a week each for maintenance, and we notice that 97 were so maintained during 1891. It would be most interesting and useful if a detailed statement of these payments were made. We notice further that 159 pay between twenty-five shillings and two guineas a-week, and the rest pay over two guineas. When referring to the visit of the Lunacy Commissioners, Dr. Philipps has the following well-put remarks: "An abrogation of the antiquated system which requires a medical commissioner to be accompanied by a legal one, and an abolition of many of the useless returns and reports ordered by the last Lunacy Act, would enable the hard-worked members of the board to give more time to the patients. When such time comes—as come it must—will disappear the unhappy misconception that safe custody, not cure, is the paramount function of asylums." The medical staff of this hospital seems ample. In addition to the Medical Superintendent there are four assistant medical officers, one of these being a lady. It is curious that no other English asylum has, as yet, followed the admirable example set here of appointing lady physicians. Dr. Philipps thinks, and we cordially agree with him, that the office of chaplain should be held by a local clergyman having a living in the neighbourhood of the asylum. H's curate would probably do the chief part of the work, and, adds Dr. Philipps, "there would be an occasional change in the personality of the officiating minister, and a change of chaplains is never undesirable."

KILLARNEY DISTRICT ASYLUM.

This asylum was opened in the year 1852, and on the last day of 1853 it contained 114 patients. It now contains 417, showing an average annual increase of eight patients. During 1891 the number of cases admitted was 117; the recoveries reached 25, and the deaths 38. The recoveries amount to 21·3 of the admissions, and, in addition, 19 were discharged improved. The deaths afford a percentage of 9·4 on the average number resident, and we notice that 13 were owing to phthisis. Additional accommodation was provided for 50 male patients, and other additions have been carried out. We notice that hot-water pipes are being placed throughout the asylum, and Dr. Griffin says this system of warming will be a source of comfort to the patients, lessen the danger from fire, and be economical in fuel. That it will do the two latter we have no doubt, but we doubt whether any method of warming approaches in comfort that of properly-constructed open fire-places, and Dr. Griffin will probably find his already high mortality from phthisis increase if he abandon them for hot-water pipes. There is no report by the Visiting Committee. The Inspector's report shows that six patients were restrained and 29 secluded, but he does not state the reason for the employment of what seems at first sight a large amount of restraint for so small an asylum. One would have thought that the recent additions to the asylum would have been likely to meet the wants of the county for a long time to come, but it is shown in this report that although the population of the country has, during the last ten years, gone down from 201,039 to 170,136, the number of lunatics has increased from 234 to 251, and the number of imbeciles from 270 to 294. This is an extraordinary state of affairs, and it would be very desirable if the Medical Superintendent and Inspector would investigate the matter and let us have in next year's report their idea of the causation of such an extraordinary fact. The asylum is apparently suffering from an uncertain water supply, and the means at hand for the extinction of fire are insufficient. There is no assistant medical officer, but one is surely needed with a population of over 400, and the omission can hardly be on the score of expense, as the visiting physician receives £100 a year, and the chemist £30. The abolition of these would provide the necessary salary. The cost per head per annum is £23 6s. 2d.

